# Reticulated Round-Cell Sarcoma of the Mediastinum, with Secondary Deposits in the Liver

**Published:** 1882-04

**Authors:** Frank V. Walton


### III.—RETICULATED ROUND-CELL SARCOMA OF THE MEDIASTINUM, WITH SECONDARY DEPOSITS IN THE LIVER.

REPORTED BY FRANK V. WALTON, D.V.S.

The dog afflicted with this disease was a fine mastiff, between two and four years of age.

In March, 1881, he suffered from a large abscess located in the right cheek. An incision was made into the tumor, and the pus allowed to escape. The remaining cavity was injected occasionally with an aqueous solution of zincichloridum gr. x., to the ℥ i. Suppuration soon ceased, and the wound healed kindly. After this the animal gained in weight and flesh, and August. 1881, weighed about one hundred and seventy-five pounds.

In September, 1881, the animal was first noticed to be losing his appetite, and shortly was unable to swallow any large masses of solid food. He also gave evidence of having great difficulty with semi-solid food, and it appeared obstructed in its passage through the œsophagus and into the stomach. From this time on he rapidly emaciated, although all forms of tonics and stimulants were administered. As his trouble advanced he experienced considerable difficulty in breathing, and had a hacking, spasmodic cough.

In December, 1881, it was noticed that the quantity of urine passed was less than it had been before; micturation became more frequent, incomplete and painful. Toward the last the urine was very scanty, the attempts to urinate very frequent and accompanied by great distress. The dog steadily sank, suffering pain, aside from the cystitis.

A necropsy was held the following day. The contents of the thorax were removed through the abdominal wall after the diaphragm was cut away. The object in operating in this way was to save the skeleton intact. The pericardial sac, both visceral and parietal, was invaded by a new growth, and it closely resembled the "hairy heart" of acute inflammation. There was no serous or purulent effusion into the pericardium.

The posterior medestinal space was completely filled with nodular masses, some of which were very soft and others moderately hard. The central point of origin of these new growths was apparently about the root of the lungs and origin of the great vessels. Large masses of the neoplasm had wedged in between the bifurcation of the trachea and the œsophagus, and in this way produced considerable pressure upon the œsophagus, which would readily explain the difficulty in swallowing during life.

The lungs were nearly normal and scarcely involved by the diseased process. There was, however, two or three very small growths in the anterior margin of the right lung. There was no striction either of the trachea or œsophagus further than that produced by the diseased masses. In the abdominal cavity the first thing noticed was an enormously distended bladder. When the bladder was cut open and the urine allowed to escape, there was a very large amount of sandy-looking sediment left upon the dependent portions of the mucous membrane. This inorganic deposit was examined microscopically at the laboratory and found to be composed chiefly of lime carbonate. The kidneys were the seat of numerous old infarctions, and had undergone a slight fatty degeneration. The liver was thickly studded with small white nodules which closely resembled, in their microscopic appearance, the tumors of the medestinal span.

Microscopic examinations of the new growths, both of the chest and liver, were made. Both presented nearly the same appearance, being composed of large round connective tissue corpuscles, which were arranged in delicate walled alveolar spaces. In both situations the tumors were very vascular, and contained many extravasations. This growth may, therefore, be regarded as sarcomatous in nature, and of the reticulated round cell variety.

New York City, 1882.

